# Adaptation of the Intelligence Structure Test, Latvian version: psychometric properties

**DOI:** 10.3389/fpsyg.2024.1319983

**Published:** 2024-03-19

**Authors:** Inese Jokste, Ingrida Trups-Kalne, Jelena Lubenko, Inga Millere, Jelena Kolesnikova

**Affiliations:** ^1^Psychology Laboratory, Riga Stradiņs University, Riga, Latvia; ^2^Aspris Wellbeing Centre, Dubai, United Arab Emirates; ^3^Faculty of Public Health and Social Welfare, Riga Stradiņš University, Riga, Latvia; ^4^Clinic of Psychosomatic Medicine and Psychotherapy, Riga Stradiņs University, Riga, Latvia; ^5^Department of Health Psychology and Paedagogy, Riga Stradiņs University, Riga, Latvia

**Keywords:** intelligence, adaptation, validity, psychometric properties, intelligence structure test (IST-2000R)

## Abstract

The Intelligence Structure Test (IST-2000R) is created to measure reasoning abilities and knowledge through verbal, numerical, and figural domains. The qualities of IST-2000R have shown its potential to be adapted and standardized in a Latvian sample to be used in psychological evaluation and research, thus satisfying the need for reliable measurement. The aim of this study was to investigate the psychometric properties of the Latvian version of IST-2000R. The adaptation sample consisted of 1,017 participants aged 15–65 (*M* = 31.8; SD = 10.94), of whom 36% were male. Participants were tested using the supervised offline administration mode (exploro.lv). The Ethics Committee of Riga Stradins University (RSU), Riga, Latvia, approved the study. The data show that the psychometric properties of the Latvian version of IST-2000R are in line with scientific norms. Thus, the test is considered to be reliable and may be used for psychological evaluation and research.

## Introduction

The ability to understand complex ideas, learn from experience, engage in reasoning, and adapt effectively to the environment are some of the traits attributable to intelligence. There are different concepts and theories of intelligence, all of which attempt to clarify the phenomenon (Van Tassel-Baska, 2005). Among these theories, there are two main approaches: so-called domain-general perspective models and domain-specific models. These two models differ mainly in understanding the dimensionality of intelligence. The domain-general perspective model considers intelligence to be a one-dimensional trait and has been described in the works of Galton, Binet, and others. The domain-specific model, on the other hand, with such authors as Thurstone, Gardener, and others, proposed that there are specific types of intelligence (Sternberg, 2020).

Another important aspect of understanding intelligence is so-called fluid and crystallized intelligence. According to Horn and Cattell (1966), fluid intelligence reflects the functioning of the central nervous system, which is the genetically determined ability to solve tasks and use analytical reasoning. Whereas crystallized intelligence is developed through experience/learning, considering also cultural background.

In the framework of the above-mentioned approaches, several intelligence measurements have been developed. Binet-Simon IQ test’s most recent version (SB5; Roid, 2003); Cattel’s Culture Fair Intelligence test (Cattell, 1940); Raven Progressive Matrices, first published in 1938, followed by renewed versions in 1940, 1956, 1998, and 2000 (Strauss et al., 2006); Wechsler’s Test of Intelligence (Wechsler, 1997). The Woodcock–Johnson Tests of Cognitive Abilities, first published in 1977 and followed by renewed versions, include both models (Woodcock et al., 2001) and, among them, the Intelligence Structure Test 2000 R (Intelligenz-Struktur-Test, IST-2000R; Liepmann et al., 2007).

There are several approaches and theories based on which the different intelligence measurements were created. Underlying the IST-2000R is Thurstone’s (1947) understanding of intelligence as consisting of multiple abilities. This basic principle is referred to as multi-trait determination of intelligence and is described in the works of Vernon (1961), Guttman (1965), Guilford (1967), Cattell (1987), and others, followed by the assumption of the proposed model as a hierarchical structure. The convergence of these two principles (Carroll, 1993) is the foundation for rationality in the development of IST-2000R (Beauducel et al., 2010).

The IST-2000R is based on Thurstone and Cattell intelligence theories, measuring verbal, numerical, and figural reasoning abilities with a composite score indicating general reasoning ability and verbal, numeric, and figural knowledge with a composite score indicating general knowledge (Liepmann et al., 2007). Based on Horn’s and Cattel’s theory of fluid and crystallised intelligence, the model describes general intelligence as consisting of two parts: fluid intelligence, which is characterized by traits of central nervous system functioning abilities, and crystallized intelligence, which is rather dependent on gained experience and cultural context (Horn and Cattell, 1966). Following Thurstone’s understanding of intelligence as a domain-specific model, IST-2000R includes verbal, numerical, and figural domains. The knowledge part, on the other hand, is not divided into task groups but consists of 84 tasks equally divided between the subgroups of verbal, numerical, and figural knowledge, and the time limit is somewhat flexible, not exceeding 40 min whatsoever (Beauducel et al., 2010).

### Approaches in psychometric properties’ evaluation for intelligence tests

Classical test theory, in the framework of which the evaluation of IST-2000R psychometric properties was done in this research, has several advantages: it is relatively easy to use in different testing situations, the mathematical procedures used for estimation are rather simple, and a relatively small sample is needed to establish the required parameter estimates (Van der Elst et al., 2013). However, it has also received some criticism for being examined-sample-dependent in terms of discrimination and difficulty indices (Bichi, 2015) and test-sample dependent on the individual achievement level (Embretson and Reise, 2000).

It must be considered that when measuring abilities, be they learning abilities or intellectual abilities, not only the population sample should be considered, but also the purpose of the test and the intended use. When testing intelligence, difficult and easy items should be included to measure the intended construct in the intended sample (Albano, 2018). No matter how the CTT approach might be criticized, it serves well to establish the different levels of difficulty for items and the discriminating ability of the items. Item analysis is usually performed by calculating the difficulty and discrimination indices (item-total correlation). As noted by Lange (1967), it is an appropriate method for deciding on the selection or rejection of the test items.

Surely, apart from the classical test theory, the Rasch model was also used as an approach for investigating different intelligence tests. For example, on gene–environment interaction and the heritability of intelligence in childhood (van Leeuwen et al., 2008), determining learning occurrence and learning rate applying computer-administered intelligence test (Verguts and De Boeck, 2000), establishing homogeneity of verbal reasoning tasks of the Intelligence Structure Test Battery (IST) (Amthauer, 1973), and others. The use of Rasch analyses in measuring abilities, including reasoning abilities, increases slowly. Still, it would be a valuable addition for further research, as it applies a statistical model predicting the mathematical relationship between an item and trait, instead of item correlation in classical test theory (Medvedev and Krägeloh, 2022). It shows a promising field of possibilities for further research of the IST-2000R Latvian version using other approaches to establish a deeper understanding of the measurements.

### Psychometric properties of Intelligence Structure Test

In the adaptation process of IST-2000R to Latvian, researchers followed the original test development procedure that includes several reliability and validity aspects, including internal consistency, split-half reliability, item analyses, and factor structure described in more detail. Calculations of difficulty and discrimination indices (item-total correlation) help to prevent researchers from being biased as to the items that would seemingly “work better” or “would be placed better” (O'Connor and Eskey, 2005). It is especially important for ability or intelligence tests that ask items to be arranged from the easiest to the most difficult, and they must be evaluated not from the experience or beliefs of the researchers but from a scientific perspective.

In the CTT framework, the item difficulty index refers to the percentage of correct responses given to the test item, which is obtained by dividing the number of correct responses by the total number of responses. In general, higher values point to the lower difficulty of the item, and vice versa; lower values point to the greater difficulty of an item (Penfield, 2013). Although it could be understood that the optimal difficulty index would be about 50% of correct responses, for most tests, the range of 0.30–0.70 is believed to be most informative regarding distinguishing between individuals. Depending on the purpose of the test, a variety of difficulty levels might be needed for the items (Kaplan and Saccuzzo, 2009). The main task of intelligence tests is to evaluate the abilities of different individuals, so there is in fact a need for items of different levels of difficulty. The main idea of the nine task groups in IST-2000R is that items start from the simpler, so the difficulty index should be rather high, and closer to the end of the task group, the items should have a rather small difficulty index. Also, it should be noted that having these items ordered from the largest to the smallest difficulty index would be advisable.

The discrimination index (item-total correlation) is another important analysis to test items and their ability to differentiate individuals. The discrimination index (item-total correlation) measures the difference between the percentage of tested individuals with the highest scores, the top 27% scorers, who obtained the correct response, and the percentage of those who obtained lower scores, the bottom 27%. The range between the lowest 27% and highest 27% score is considered optimal to distinguish individuals with respect to the variable measured. Higher discrimination indices (item-total correlation) show a better ability to determine the difference, or discriminate, between those individuals with high test scores and those with low ones. It is also important to notice the connection between the difficulty index and discrimination index (item-total correlation), and it must be noted that there is a general rule—as the difficulty of an item increases, discrimination approaches zero (O'Connor and Eskey, 2005).

### Reliability

Cronbach’s alpha coefficients were calculated to determine the internal consistency of the Intelligence Structure Test. A value of 0.7–0.8 is considered an acceptable value for Cronbach’s alpha for most measurements; accordingly, values substantially lower indicate an unreliable scale (Field, 2017). Furthermore, as was noted by Kline (1999), generally, Cronbach’s alpha of ≥0.8 would be considered to be a good fit, but values of 0.8 and above are more appropriate for cognitive tests, including intelligence tests.

As IST-2000R has been adapted to several languages, it is important to see and compare Cronbach’s alpha for these versions. The reasoning part of IST shows appropriate Cronbach’s alpha values for intelligence tests, according to Kline (1999): the original German version from 0.72 (verbal reasoning) to 0.94 (numerical reasoning) (Bühner et al., 2006); the English version from 0.86 (verbal reasoning) to 0.95 (numerical reasoning) (Beauducel et al., 2010); and the Lithuanian version from 0.93 (figural reasoning) to 0.98 (numerical reasoning) (Šimelionienė and Gintilienė, 2011). However, Cronbach’s alpha values for the Italian version show appropriate values according to Field (2017) for verbal reasoning (0.70) and numerical reasoning (0.70), but weak values for figural reasoning (0.41) (Pellerone et al., 2015). It shows that in all versions, the reliability of numerical reasoning is the strongest.

The analysis was then continued with the split-half reliability procedure, which provides another measure of reliability. The items are randomly split into two parts, and the score of each part is obtained. To establish split-half reliability, the parts are then correlated. Reliability is interpreted as a correlation coefficient, where high correlations would show strong reliability (Kline, 1999). The results for the English version range from 0.60 to 0.92 for task groups, the lowest for sentence completion and highest for 0.92 number series; and from 0.87 to 0.92 for subscales, for a reasoning total of 0.96 (Beauducel et al., 2010). For the Lithuanian version, from 0.84 to 0.96, the lowest for verbal similarities and the highest for number series, reasoning total of 0.97 (Dragūnevičius and Gintilienė, 1998).

### Construct validity

There are two ways factor analysis is used: confirming the proposed structure (confirmatory factor analyses) and investigating the structure (exploratory factor analyses) (Olkin and Sampson, 2001). In cases where there is no specific theory on how many latent factors are present in the measurement and the relationship between them is not clear, exploratory factor analysis (EFA) is a helpful tool for investigating the number of latent factors and the relationship between them (Shou et al., 2022). When there is an available EFA analysis for the instrument, confirmation factor analysis greatly contributes to understanding the measurement by comparing it with the existing theoretical model and thus confirming its fit or finding an unfitting factor structure (Tavakol and Wetzel, 2020).

#### Dimensionality and differential item functioning

In psychometrics, the validation of the model often assumes that unobservable constructs are created from observed variables, where the constructs are compared across different groups (e.g., male and female), assuming that the constructs are invariant (Sass and Schmitt, 2013). When comparing scalar and metric models, if the overall model’s fit does not appear to be significantly worse, it may be assumed that items across the groups do not significantly impact the model fit (Putnick and Bornstein, 2016). However, this is not always accurate, and there is a need to assess the unidimensionality by checking whether the general factor is strong enough to assume sufficient unidimensionality (McDonald, 1999). An item or test score is considered unidimensional if the systematic differences within the item variance are only due to one latent variable. Test scores could also be multidimensional, as test items may measure more than one psychological process (Bejar, 1983), and in such cases, the test should be able to reflect those processes (Ziegler and Hagemann, 2015). To test the unidimensionality of items, several techniques could be used, including exploratory factor analyses (EFA), confirmatory factor analyses (CFA), and item response techniques (IRT), each with its own advantages (Ziegler and Hagemann, 2015). Marcoulides and Yuan (2017) have proposed a range of adjectives associated with certain values of the RMSEA (0.01 = “excellent,” 0.05 = “close,” 0.08 = “fair” and 0.10 = “poor”) and the CFI (0.99 = “excellent,” 0.95 = “close,” 0.92 = “fair” and 0.90 = “poor”), which might be helpful while describing the goodness of model fit.

Differential item functioning (DIF) analysis means examining responses given to the items to establish whether these responses differ because of sex, age, native language, or other aspects, and to make sure that everyone is given equal probability to respond to the item according to their true ability levels. This analysis can be performed by calculating various statistics, including Mantel–Haenszel, which can be carried out using jMetrik (Annan-Brew, 2021).

In the analyses, each item is classified as having either A-, B-, or C-level DIF. Items with A-level DIF are considered to be a good fit item. B level points to moderate level of DIF and C-level items to large level of DIF. A-level DIF is applicable if (a) the chi-square statistic is <3.84 and the value of *p* >0.05 or (b) the common odds ratio is between 0.65 and 1.53. Items with B-level DIF are seen as questionable, and to establish B level, the chi-square statistics, value of p, and common odds ratio should differ from both A and C levels. For items with C-level DIF, (a) the common odds ratio is <0.53 and the upper bound of the 95% confidence interval for the common odds ratio is <0.65, or (b) the common odds ratio is >1.89 and the lower bound for the 95% confidence interval for the common odds ratio is >1.53 (Meyer, 2014).

## Method

The research to form a culturally appropriate and psychometrically correct Latvian version of IST-2000R had two phases.

### The first phase

#### Measurements

Sociodemographic questionnaire: sex, age, education level, and native language.

The first Latvian language version of IST-2000R (Beauducel et al., 2010) was prepared by the research team. The reasoning part of IST-2000R consists of verbal, numerical, and figural tasks. Sentence completion, verbal analogies, and similarities are verbal task groups; calculations, number series, and numerical signs are numerical task groups; and figure selection, cubes, and matrices are figural task groups. Together, there are nine task groups, each consisting of 20 tasks.

#### Participants

In the first phase of the adaptation of the Latvian version of the IST-2000R, there were 266 participants aged 16–67 (M = 27.5; SD = 10.94). Of them, 61.7% were female. For 94% of the participants, Latvian is the native language or is used daily in communication within the family. 22.9% are still at school, 15.85 have secondary education, 5.6% have vocational education, 15.8% are university students, and 39.8% have higher education.

#### Procedure

For adaptation purposes, the English version of IST-2000R was used (Beauducel et al., 2010). Detailed psychometric properties of the test can be found in the IST English version Manual (Beauducel et al., 2010), and the main indices are shown in the result part of this article, compared to the data of the Latvian sample. Latvian translations were made of all instructions included in the test booklet and standardized protocols for test administrators. The translation was done by two professional translators after several psychology experts with appropriate English knowledge reviewed the translation. The test administrators then underwent a short training. Training included an introduction to the theoretical basis of IST-2000R, its structure, and standardized testing procedure. This was specifically done to ensure that the data were gathered in a standardized way. During the training, the purpose of the research—establishing the psychometric properties of the Latvian version of IST-2000R—was also explained.

In the first phase, the first Latvian version of the IST-2000R test was used. The research took place in April and May 2019. Participants were invited to participate in this research *via* the RSU webpage, which was advertised on social networks and mass media. The sample was formed based on convenience and snowball principles. Testing was carried out using the paper–pencil method both individually and in groups, which is supported by the administration principles of IST-2000R. Testing was supervised by trained test administrators and psychologists.

Based on the results of the first phase of the research, the necessary adjustments were made to some verbal reasoning items for which discrimination indices (item-total correlation) were out of the normal range—negative or lower than 0.2. Such results show that items do not differentiate individuals according to the trait that is being measured.

To improve the psychometric properties of verbal scales, an expert group was formed to investigate the face validity of items with too low discrimination indices (item-total correlation). This was done by interviewing participants of different age groups and different educational levels and asking about the thought process and the reasons for choosing the answer they chose as the correct one. At the same time, consultations with experts in different scientific fields helped to work out the best and most precise options for translating answer choices (distractors) from English into Latvian. The corrected items were then tested in a pilot study (*n* = 30) and included in the second Latvian version of IST-2000R.

Changes were made in 18 items: nine in sentence completion, six in verbal analogies, and three in verbal similarities. In addition, based on the results of the difficulty indices, the order of some items within the groups was changed. In the numerical and figural task groups, neither items nor their order within the groups were changed.

There were three types of adjustments for items following the first phase of the research: a more precise form of item and/or distractor translation; the replacement of a distractor; and the full replacement of the item.

An example of more precise item translation would be items from verbal analogies. The English version of the item was “Nerve: line = pupil:…? (a) sight (b) eye (c) shield (d) radiation (e) light” which was translated into Latvian exactly in the same way. However, the difficulty index and discrimination index (item-total correlation) were too low, at 0.16 and 0.26, respectively. Looking into the German version of the same item, “Leitung = Pupille:? (a) Sehen (b) Auge (c) Blende (d) Strahlung (e) Licht,” instead of “shield” the word “Blende” is used, which translates as “photo diaphragm.” As the translation from German more precisely corresponds to the analogy used in this task group, it was decided to use the translation from the German version further. After the adjustments, the difficulty index (0.32) and discrimination index (item-total correlation) (0.29) improved.

In the task group of verbal similarities, the replacement of distractor was used for one of the items. The answer was translated from English (the German version is the same): “(a) infarct (b) Aids (c) scurvy (d) flu (e) polio (f) diabetes” and the task is to find two words that are meaningfully connected. During the first phase of the research, the difficulty index and discrimination index (item-total correlation) were too low, at 0.03 and 0.10, respectively. As “scurvy” is a disease that is rare and barely known for many, the alternative distractor was chosen—“anemia,” which, similarly to “scurvy,” describes the state of a significant deficit of a nutrient (in the case of “scurvy”—vitamin C; “anemia”—iron). After the replacement of the distractor difficulty index and discrimination index (item-total correlation), they slightly improved to 0.12 and 0.19, respectively, and as this item is the last in the task group, it is meant to be difficult, so these results can be acceptable.

And finally, there was a case of a full replacement of the item in the sentence completion task group. Item “The opposite of comfort is…? (a) disappointment (b) mitigation (c) discouragement (d) despair (e) suppression.” The word “discouragement” is not easy to translate into Latvian, and it would be either a two-word phrase like “losing one’s courage” or “demotivation.” Using “demotivation” did not show good results. So, it was decided to replace the item with “The opposite of ‘balance’ is…? (a) zero gravity, (b) vacuum, (c) chaos, (d) equilibrium, (e) anarchy,” which has similar principles as the original item. After the replacement difficulty index (0.52) and discrimination index (item-total correlation) (0.75).

It must be noted that all corrections were communicated and coordinated with the holders of the ownership rights of the test—Hogrefe Publishing Group.

### The second phase

#### Measurements

Sociodemographic questionnaire: sex, age, education level, and native language.

The Latvian version of IST-2000R (Ļubenko et al., 2022) (second version). The reasoning part of the IST-2000R Latvian version consists of 180 items divided into three parts: verbal, numerical, and figural reasoning, each consisting of three task groups in which 20 items are included. In each of the three parts, there are five specific stimuli (verbal, numerical, or figural) as the possible answer choices to the task given. Only one of those choices is the most precise, and the other four are distractors. The chosen answer is considered right or wrong, making this a dichotomous scale.

#### Participants

In the second phase of the research, there were 1,017 participants aged 15–65 (*M* = 31.8; SD = 12.67), of whom 36.2% were male. Latvian was the native language for 86% of participants, 13% mentioned Russian, 0.4% mentioned other languages, and 13% did not mention their native language. The sample consisted of several age groups: 15–18 (13.85%), 19–20 (10.2%), 21–25 (14.15%), 26–30 (16.1%), 31–40 (21.9%), 41–50 (14.0%), and 10% were 50 years old and older. In the age group 15–18, 69% already had basic education or lower, 13% had vocational education, and 18% had secondary education. In the age group 19–20, 8.7% had basic education, 21.2% had secondary vocational education, 67.3% had secondary education, and 2.8% had higher education. In the age group 21–25, 3.5% had basic education, 11.8% had secondary vocational education, 48.3% had secondary education, and 36.4% had higher education. In the age group 26–30, 3.7% had basic education, 11.1% had secondary vocational education, 21.6% had secondary education, and 63.6% had higher education. In the age group 31–40, 4.6% had basic education, 11.4% had secondary vocational education, 14.6% had secondary education, and 69.4% had higher education. In the age group 41–50, 2.9% had basic education, 16.8% had secondary vocational education, 8.0% had secondary education, and 69.4% had higher education. In the age group 50 years and older, there were no participants with only basic education; 20.8% had secondary vocational, 18.8% had secondary, and 50.4% had higher education.

#### Procedure

The Ethics Committee of Riga Stradins University (RSU), Riga, Latvia, approved the study.

The sample for the second phase of the research was formed according to the principles of the stratified sample, with the aim of including participants from all regions of Latvia as well as aiming for a specific number of participants in each age group. Participants were recruited for the research through the RSU website, social networks, media, TV, and radio interviews with the researchers.

When applying, participants read and signed the participant’s informed consent form for participation in the study and were able to choose the place and time of testing. Before testing, the study participants received information about the study. When testing participants younger than 18 years old, the consent of parents or legal guardians was obtained. The testing took place at RSU and universities, schools, and libraries in different regions of Latvia and was administered by psychologists who had undergone previous training to ensure the standardized testing procedure (see Procedure in the first phase of the research) and were assisted by psychology students of RSU. Exploro Ltd. created the computerized version of IST-2000R for the needs of the second phase of the study.

Data were collected during March–October 2020, considering COVID-19 recommendations. The duration of the test for the reasoning part was 1 h and 20 min. Computerised testing was carried out in small groups (up to 15 people). After completing the test, the respondents received brief feedback on their results by email. The RSU Psychology Laboratory conducted the adaptation research process of the IST-2000R Latvian version in Latvia.

## Results

To have an overall understanding of the results of the IST-2000R Latvian version test, the descriptive statistics are presented in Table 1. These results are from the second phase of the research.

**Table 1:** Descriptive statistics of the reasoning part of the IST-2000R Latvian version.

	*M*	*SD*	Skewness	Kurtosis	Minimum	Maximum
Sentence completion	12.16	3.65	−0.52	−0.19	0.00	20.00
Verbal analogies	10.83	4.35	−0.42	−0.81	1.00	19.00
Verbal similarities	10.99	4.63	−0.63	−0.60	0.00	20.00
Calculations	11.48	4.92	−0.11	−0.82	0.00	20.00
Number series	9.87	5.91	0.13	−1.15	0.00	20.00
Numerical signs	12.24	4.93	−0.46	−0.89	0.00	19.00
Figure selection	10.84	4.12	−0.16	−0.51	0.00	20.00
Cubes	10.39	3.84	−0.12	−0.29	0.00	20.00
Matrices	9.68	3.44	−0.33	−0.32	0.00	19.00
Verbal reasoning	33.96	11.17	−0.63	−0.51	1.00	56.00
Numerical reasoning	33.48	13.66	−0.21	−0.81	2.00	59.00
Figural reasoning	30.86	9.08	−0.24	−0.27	0.00	54.00
Reasoning total	98.12	30.13	−0.52	−0.37	12.00	158.00

The reliability of the IST-2000R Latvian version reasoning part was evaluated by Cronbach’s alpha and split-half reliability. Cronbach’s alpha for task groups ranged from 0.74 to 0.90. The lowest were for sentence completion and matrices, and the rest were above or nearly above (for example, figure selection *a* = 0.079), which is advisable for intelligence tests according to Kline (1999). The results for the subgroups were verbal *a* = 0.92, numerical *a* = 0.96, and figural *a* = 0.88. The total Cronbach’s alpha for the reasoning part was 0.97.

The split-half reliability ranges from *r* = 0.60 to *r* = 0.92 (*p* < 0.001), showing moderate to strong correlations between items in task groups. The strongest correlation is for number series (*r* = 0.92), and the weakest, but still acceptable, is for sentence completion (*r* = 0.60). For the subgroups, the results were as follows: verbal *r* = 0.88, numerical *r* = 0.95, and figural *r* = 0.84. For the total reasoning part, it was *r* = 0.96 (see Table 2).

**Table 2:** Psychometric properties of the IST-2000R Latvian version.

	Cronbach’s alpha LV (ENG)	Split-half correlation LV (ENG)	Kendall’s Tau correlation LV (ENG)	Range of difficulty indices	Range of discrimination indices (item-total correlation)
Verbal reasoning	0.92 (0.86)	0.88 (0.87)	—	—	—
Sentence completion (SC)	0.74	0.60	−0.72	0.40–0.89	0.13–0.79
Verbal analogies (VA)	0.83	0.72	−0.86	0.16–0.94	0.25–0.89
Verbal similarities (*VS*)	0.86	0.78	−0.86	0.12–0.86	0.19–0.90
Numerical reasoning	0.96 (0.95)	0.95 (0.96)	—	—	—
Calculations (CA)	0.90	0.85	−0.90	0.18–0.98	0.05–0.95
Number series (NSe)	0.93	0.92	−0.91	0.12–0.94	0.19–0.96
Numerical signs (NSi)	93	87	−0.96	0.01–0.99	0.00–0.95
Figural reasoning	0.88 (0.88)	0.84 (0.89)	—	—	—
Figure selection (FS)	0.79	0.77	−0.58	0.21–0.77	0.35–0.76
Cubes (CU)	0.80	0.77	−0.67	0.05–0.79	0.05–0.84
Matrices (MA)	0.74	0.62	−0.68	0.12–0.91	0.15–0.64
Reasoning	0.97 (0.95)	0.96 (0.96)	—	—	—

The items in each task group are arranged in accordance with their difficulty level—each task group starts with easier items and finishes with the most difficult, except figure selection and cubes, where, because of the little shift in the stimuli, there is a tendency for items to become more difficult toward the middle of the task group and then pick up from more easy ones to most difficult again. In the numerical and figural subgroups, the placement of items was not changed; it was the same as in the English (and German) versions. In the verbal subgroup, however, the items were placed in order according to the data from the first phase of the study, so the placement of the items is not the same as in the English and German versions (and some of the items were changed as well, adjusting them to the needs of language and culture).

The item difficulty indices are shown in Table 2. As the Intelligence Structure Test items are dichotomous—coded as 0 or 1—the index range varies between 0 and 1. It shows how many of the participants in the research have chosen the correct answer.

Kendall’s Tau correlation coefficients between the difficulty indices of the items and the sequence number of the items in each task group show the gradual increase in difficulty within each task group. The correlation coefficient should be negative and as close to −1 as possible, which would show that the difficulty of the item grows according to the sequence of the item.

Kendall’s Tau coefficients for IST task groups range from −0.72 to −0.86 for verbal reasoning, from −0.90 to −0.96 for numeral reasoning, and from −0.58 to −0.68 for figural reasoning (see Table 2). The most appropriate item sequence is found in numerical reasoning task groups, where the items in task groups start from the easiest and finish with the most difficult tasks (see Appendix Figure A3). In figure reasoning task groups, it is rather typical to have a sequence of item difficulty to start from item 1 and gradually increase at item 10, then there is a slight change in the task stimuli, and item 11 tends to be easier, and the following items gradually increase in difficulty level u to item 20 (see Appendix Figure A3). In verbal task groups, there are some inconsistencies in the item sequence and item difficulty level. However, in general, there is quite a good tendency for the difficulty level to grow from easier items at the beginning of the task group to the most difficult at the end (see Appendix Figure A2).

The range of discrimination indices (item-total correlation) is shown in Table 2. The results for verbal reasoning task groups range from 0.19 to 0.90, for numerical reasoning task groups from 0.00 to 0.96, and for figural reasoning task groups from 0.05 to 0.84 (see Table 2). There are several items that fall out of the normal range (0.20–0.80). However, it must be noted that intelligence test items should vary widely in terms of difficulty to be able to measure very low intellectual abilities with a difficulty index above 0.8 as well as very high intellectual abilities with a difficulty index below 0.2. Such a tendency in these data shows that there are a number of participants that were able to deal only with the easiest tasks and some that were able to deal with the most difficult tasks.

Table 3 shows correlations between task groups, providing evidence for the positive relationship of reasoning task groups as measuring the same construct—reasoning altogether and verbal, numerical, and figural reasoning when looking at correlations between specific task groups. So for verbal reasoning tasks, correlations range from 0.63 to 0.69; for numerical reasoning tasks, correlations range from 0.55 to 0.75; and for figural reasoning tasks, from 0.43 to 0.55. For reasoning altogether, correlations range from 0.27 to 0.75, showing evidence of a close relationship between different task groups. The results are similar to the results of the English version (Beauducel et al., 2010).

**Table 3:** Correlations between task groups.

Task group	SC	VA	VS	CA	NSe	NSi	FS	CU	MA
Sentence completion (SC)		0.68	0.63	0.54	0.46	0.54	0.51	0.27	0.47
Verbal analogies (VA)	0.52		0.69	0.63	0.51	0.63	0.56	0.31	0.54
Verbal similarities (*VS*)	0.47	0.59		0.53	0.45	0.54	0.49	0.27	0.49
Calculations (CA)	0.34	0.49	0.44		0.55	0.75	0.54	0.37	0.50
Number series (NSe)	0.30	0.45	0.41	0.61		0.58	0.46	0.36	0.50
Numerical signs (NSi)	0.34	0.45	0.44	0.69	0.63		0.56	0.41	0.57
Figure selection (FS)	0.27	0.39	0.42	0.40	0.43	0.49		0.43	0.55
Cubes (CU)	0.20	0.29	0.30	0.32	0.38	0.42	0.54		0.44
Matrices (MA)	0.25	0.39	0.35	0.32	0.43	0.42	0.48	0.48	

The lower triangle of the matrix—Great Britain sample: *N* = 1894, the upper triangle of the matrix—Latvia sample: *N* = 1019.

## Confirmatory factor analyses

**Figure 1:**
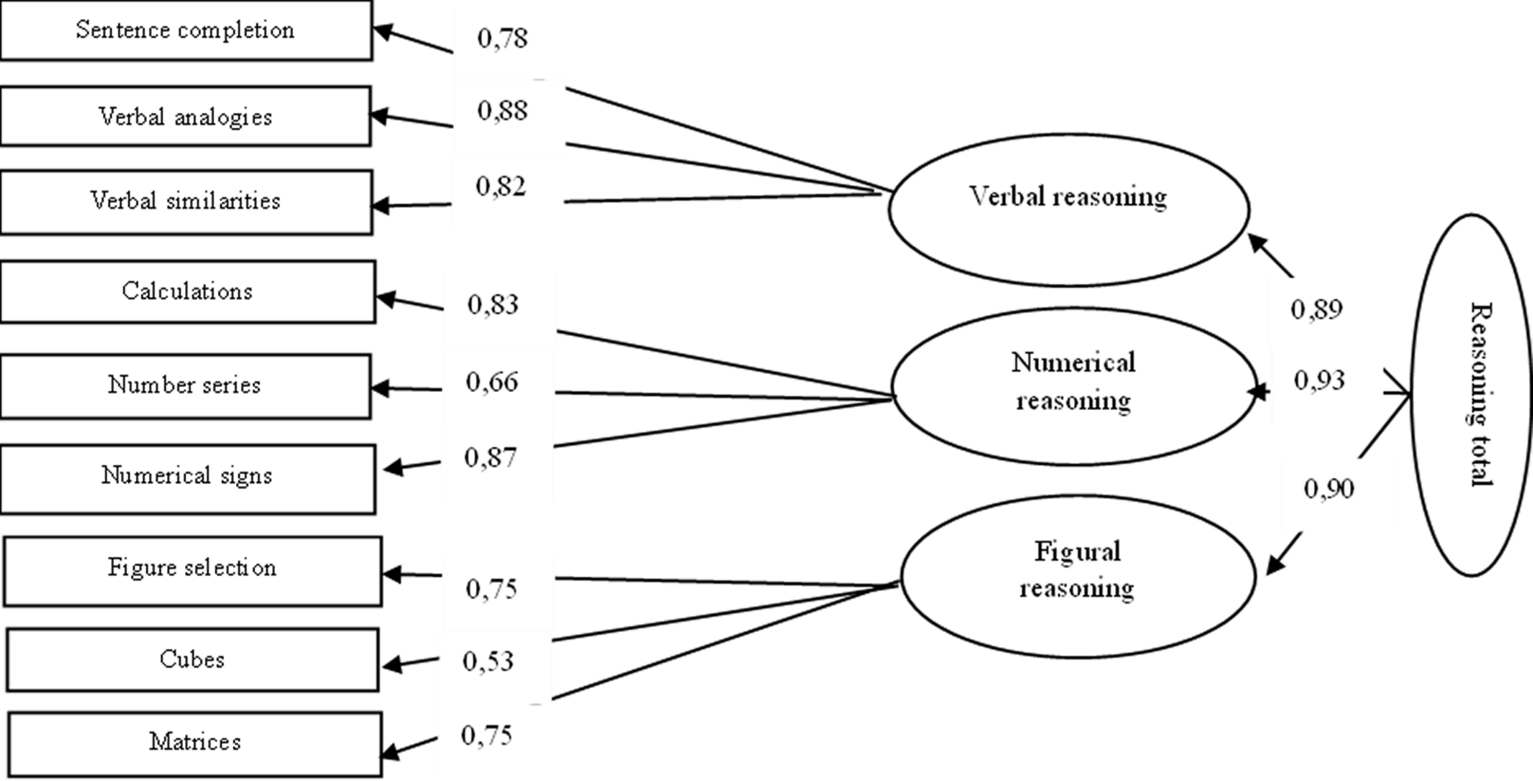
Confirmatory factor analysis model for reasoning part of the IST-2000R Latvian version.

An investigation of the structure of the reasoning part model of IST-2000R (Latvian version) was carried out using confirmatory factor analysis using *RStudio* (*N* = 1,017). Lavaan was used for maximum likelihood (ML) confirmatory factor analyses (CFA) of the nine reasoning part aggregates. Three reasoning content factors (verbal, numerical, and figural) and a total reasoning factor were postulated. The model fits quite well with the data (see Figure 1). The chi-square value is 80.435 (df = 24; *p* < 0.001). The goodness-of-fit index (GFI) value is 0.98, and the adjusted goodness-of-fit index (AGFI) value is 0.96. The root mean square residual (RMR) value is 0.50, and the standardized root mean square residual (SRMR) value is 0.027. The comparative fit index (CFI) value is 0.988. The root mean square error of approximation (RMSEA) value is 0.048. Overall, the model fit appears to be acceptable.

When comparing the results of CFA to the English version of IST-2000R, based on which the translations for adaptation were made, we can see pretty similar results (see Table 4).

**Table 4:** Confirmatory factor analysis for the nine reasoning tasks group.

Task group	Verbal reasoning	Numerical reasoning	Figural reasoning
Sentence completion	**0.78** (0.62)		
Verbal analogies	**0.88** (0.80)		
Verbal similarities	**0.82** (0.75)		
Calculations		**0.83** (0.80)	
Number series		**0.66** (0.76)	
Mathematical signs		**0.87** (0.84)	
Figure selection			**0.75** (0.77)
Cubes			**0.53** (0.68)
Matrices			**0.75** (0.66)

Comparison of the Latvian and English versions of IST-2000R. The bold values is Latvian.

## Dimensionality analyses

The unidimensional model was checked for each of the nine test groups. The analyses were done using CFA with a DWLS estimator.

The results for the sentence completion task group show a chi-square value of 227.702 (df = 170). The goodness-of-fit index (GFI) value is 0.974. The comparative fit index (CFI) value is 0.988, showing a close relative fit. The standardized root mean square residual (SRMR) value is 0.061, and the root mean square error of approximation (RMSEA) value is 0.019, showing a close absolute fit. However, two of the items seem to have low factor loadings; when checking the model without those two items, it improved slightly (SMRS = 0.060; CFI = 0.990). Both of those items had very low discrimination (item-total correlation) indices, and both seemed to assume more general everyday knowledge than verbal reasoning.

Verbal analogies task group shows the chi-square value is 210.767 (df = 170). The goodness-of-fit index (GFI) value is 0.985. The comparative fit index (CFI) value is 0.996, showing excellent relative fit. The standardized root mean square residual (SRMR) value is 0.055. The root mean square error of approximation (RMSEA) value is 0.016, showing a close absolute fit.

Verbal similarities task group shows the chi-square value is 251.595 (df = 170). The goodness-of-fit index (GFI) value is 0.989. The comparative fit index (CFI) value is 0.996, showing excellent relative fit. The standardized root mean square residual (SRMR) value is 0.057. The root mean square error of approximation (RMSEA) value is 0.022, showing a close absolute fit. No issues with the items were found for the verbal analogies or verbal similarities task groups.

The results for the calculations task group from the Numerical Reasoning Scale show the chi-square value is 549.349 (df = 170). The goodness-of-fit index (GFI) value is 0.987. The comparative fit index (CFI) value is 0.990, showing excellent relative fit. The standardized root mean square residual (SRMR) value is 0.090. The root mean square error of approximation (RMSEA) value is 0.048, showing a close absolute fit. All items show rather high factor loadings.

Number series task group shows the chi-square value is 1053.996 (df = 170). The goodness-of-fit index (GFI) value is 0.991. The comparative fit index (CFI) value is 0.992, showing excellent relative fit. The standardized root mean square residual (SRMR) value is 0.095. The root mean square error of approximation (RMSEA) value is 0.074, showing a fair absolute fit. In this case, too, the factor loadings are rather high.

Numerical signs task group shows the chi-square value is 841.72 (df = 170). The goodness-of-fit index (GFI) value is 0.981. The comparative fit index (CFI) value is 0.983, showing a close relative fit. The standardized root mean square residual (SRMR) value is 0.123. The root mean square error of approximation (RMSEA) value is 0.064, showing a fair absolute fit. Factor loadings are rather high for this task group (above 0.5), except for one item. When excluding this item, the absolute fit slightly improves (SRMR = 0.101; RMSA = 0.064), but not enough. It must be noted that the item is the last one in the task group, meaning that it is meant to be difficult to solve, and also that the time limit might restrict individuals’ ability to give the correct answer.

Figure selection task group shows the chi-square value is 639.407 (df = 170). The goodness-of-fit index (GFI) value is 0.924. The comparative fit index (CFI) value is 0.923, showing a fair relative fit. The standardized root mean square residual (SRMR) value is 0.088. The root mean square error of approximation (RMSEA) value is 0.054, showing a close absolute fit. The peculiarity of this task group is a slight stimulus change in the middle of the task group, and there is a tendency for item difficulty to decrease toward item 128 and increase again afterwards.

Cubes task group shows the chi-square value is 2273.856 (df = 170). The goodness-of-fit index (GFI) value is 0.883. The comparative fit index (CFI) value is 0.857, showing a poor relative fit. The standardized root mean square residual (SRMR) value is 0.114. The root mean square error of approximation (RMSEA) value is 0.175, showing a poor absolute fit. One of the items shows very low factor loading, but even excluding this item does not improve the results for them to be acceptable. The item in question is again one of the last items, and it is expected to be difficult. Also, the time limit might have affected the number of correct items given.

Matrices task group shows the chi-square value is 791.165 (df = 170). The goodness-of-fit index (GFI) value is 0.913. The comparative fit index (CFI) value is 0.856, showing a poor relative fit. The standardized root mean square residual (SRMR) value is 0.118. The root mean square error of approximation (RMSEA) value is 0.062, showing a fair absolute fit. In this case, there are two items with very low factor loadings; excluding them, the fit indices improve (CFI = 0.919; RMSEA = 0.053). Again, the two items in question are the last ones in the task group, and the same considerations apply.

## Differential item functioning

Additionally, the model was tested for dimensionality on a categorical variable—sex. According to Chen (2007), invariance level is rejected if, comparing the models, RMSEA increases by ≥0.010–0.015 and CFI decreases by ≤0.005–0.010. In our case, RMSEA decreases from 0.048 to 0.046 and CFI increases from 0.988 to 0.989, meaning the invariance should not be fully rejected. Altogether, the scalar model shows the chi-square value is 96.518 (df = 48; *p* < 0.001). The goodness-of-fit index (GFI) value is 0.978. The standardized root mean square residual (SRMR) value is 0.028. The comparative fit index (CFI) value is 0.988. The root mean square error of approximation (RMSEA) value is 0.048. Overall, the model fit appears to be acceptable.

To establish the presence of DIF, Mantel–Haenszel analyses (jMetrik) for each item were done using sex as a dependent variable. From all 180 items in IST, five items exhibited C levels of DIF and 22 items exhibited B levels of DIF, showing a large and moderate amount of DIF. All the C levels of DIF were found in the verbal subscale; from that, one item (item 14) exhibited DIF for male and four (items 3, 30, 44, and 46) for female, respectively.

Item 14 from the sentence completion task group includes a description of a mechanism. As such, it might be understood better by male than female. The difficulty index also points to the fact that it is 0.45 for male and 0.29 for female. However, the mechanism in question is a simple, everyday mechanism that does require a deep understanding of mechanics. The item-total correlation is almost the same for male (0.35) and female (0.36).

The content of item 3 includes a description of clothing, which might be better understood by female. However, it is one of the easiest items, with a difficulty index of 0.87 for male and 0.92 for female. The item-total correlation is 0.32 for male and 0.37 for female.

Item 30 content-wise does not seem really arbitrable to any of the genders specifically. The item is from task group verbal analogies, where one must find an analogy that is in accordance with the analogy given in the task “chronic: acute = constant:…?” The item-total correlation is 0.38 for male and 0.51 for female. The difficulty index is 0.45 for male and 0.55 for female.

Items 44 and 46 are both from the verbal similarities task group, where one must identify which two of six given words make a pair that classifies most precisely. In both cases, the correct answers include closing items, which might be more easily classified as female. The item-total correlation for item 44 is 0.63 for male and 0.57 for female and the difficulty index is 0.74 for male and 0.83 for female. The item-total correlation for item 46 is 0.71 for male and 0.62 for female, and the difficulty index is 0.68 for male and 0.76 for female.

There are 11 items with B-level DIF for verbal reasoning tasks. Two of these items, in favor of male, include chemistry and geography content. The rest, those in favor of female, include content about architecture, nature, travel, medicine, etc.

Numerical reasoning tasks operate only with numbers and calculations using logic, and there is no verbal context; however, there are also seven B-level DIF items, one of which is in favour of female.

Figural reasoning tasks include only shapes, forms, and patterns. However, four B-level DIF items were also found there, one of which was in favor of female.

## Discussion

The aim of the research was reached, and the Intelligence Structure Test was adapted for use within the Latvian culture and language context. The reliability of the Latvian IST version was tested by evaluating Cronbach’s alpha and split-half reliability; psychometric properties were tested by item discrimination index (item-total correlation), item difficulty index, and Kendall’s Tau; as well as confirmatory factor analyses were performed to prove factor validity; additionality was investigated by investigating the unidimensionality and the presence of DIF items. The research results were analyzed according to the principles of classical test theory and compared with the results of the Intelligence Structure Test adaptation into English, Lithuanian, and Italian languages.

Internal consistency for all task groups was acceptable, and for most of them, except Sentence Completion and Matrices (*a* = 0.74), it reached over 0.80, which is advisable for intelligence tests, according to Kline (1999). Furthermore, the appropriate reliability was supported by the results of the split-half reliability method, showing that the correlations between the items in the task groups ranged from moderate to very strong. The sentence completion task group showed the weakest of all test groups, but still a rather strong correlation. Similarly, very strong evidence for reliability is found in the German (Bühner et al., 2006), English (Beauducel et al., 2010), and Lithuanian (Šimelionienė and Gintilienė, 2011) versions of the Intelligence Structure Tests. For the Italian version, however, Cronbach’s alpha values show appropriate values, according to Field (2017), for the verbal reasoning (0.70) and the numerical reasoning (0.70), but are weak for the figural reasoning (0.41) (Pellerone et al., 2015).

Verbal reasoning tasks had the most work put in, as the items carry not only language but also cultural context. Translation into Latvian was rather challenging while trying to keep items as identical to the original version as possible and as Latvian language and culture appropriate as needed to be understood correctly. Items in verbal reasoning tasks are mostly arranged according to difficulty. However, the items were placed in order according to the data from the first phase of the study, so the placement of the items is not the same as in the English and German versions. Some of the items were adjusted to the specificity of the Latvian language and culture. The range of discrimination indices (item-total correlation) for verbal reasoning task groups show appropriate abilities to distinguish individuals with higher and lower ability levels. Also, in verbal task groups, there seem to be some slight inconsistencies in the item sequence and item difficulty level, but in general, there is quite a good tendency for the difficulty level to increase from easier items at the beginning of the task group to the most difficult at the end (see Picture 1).

In numerical reasoning tasks, no changes were made following the study’s first phase. These task groups seem to have the most appropriate item sequence, where the items in the task groups start with the easiest and finish with the most difficult tasks. However, it must be noted that in these task groups, the discrimination indices (item-total correlation) do fall out of range, and some items were not answered correctly by anyone or participants did not have enough time. It is mostly attributed to the calculation task group and the numerical signs task group (see Picture 2).

In figural reasoning tasks, no changes were made following the pilot study. However, there is a rather interesting tendency for the sequence of item difficulty to start from item 1 and gradually increase at item 10, then there is a slight change in the task stimuli, and item 11 tends to be easier again, and following items gradually increase in difficulty level up to item 20 (see Picture 3). However, it does not affect the discrimination abilities of the task group very much.

As to the factor structure of the Intelligence Structure Test, it was evaluated by confirmatory factor analyses using *RStudio*. Results generally indicate an acceptable model fit, as it is in the English version (Beauducel et al., 2010). However, checking the model for unidimensionality, a few issues were found for some task groups in numerical and figural reasoning subscales; the invariance cannot be fully rejected, and additionally, the Mantel–Haesnze method for differential item functioning clearly shows that from 180 items in IST, five items have C levels of DIF and 22 items have B levels of DIF. All C-level DIF items are found in verbal reasoning tasks; some of them might be explained by typical sex differences, but not all. None of these items were excluded from the test, as it would impact the integrity of the measurement. However, intelligence tests in general are revised more often than any of the other tests, as they at least partly depend on changes in the cultural context of society. As an example, such a simple item as a mechanical clock might lose its place in everyday life’s context as electronic devices become more and more used for reading time. Such items might become less relevant in the future. The existing version of the Intelligence Structure Test Latvian version shall be revised in the foreseeable future, and the knowledge of unidimensionality and DIF-level items will be integrated into the study.

Some limitations are still present in this research, and those should be considered in continuing work with the Latvian version of the Intelligence Structure Test. First, only the factorial validity was described in this article; however, it would be valuable to check the convergent validity by comparing results with other intelligence tests. The current research was carried out within the framework of classical test theory; however, there is enough evidence that using the Rasch modeling approach would be beneficial to get a more precise insight into the item–latent factor relationship. Another field of more specific research would be not just the general population but specifically targeted individuals with presumably a bit higher and a bit lower than average level of intelligence, to get insight into the group specifics and possibly to prove existing strategized norms or the necessity to change those. As described above, participants were mainly Latvian as their native language; however, some of them were bilingual (about 13.4%), which could have affected the results of this investigation, specifically in verbal task groups.

In this article, only part of the greater research is described; it also involved the adaptation of the knowledge part of the test as well as the standardization of both parts. As the Latvian version of the Intelligence Structure Test is not only adapted but also standardized (Ļubenko et al., 2022), it would be of use to a wide range of specialists, starting from psychologists in private practices and school psychologists, to help students find out their strengths and weaknesses, for organizations, and psychologists working for organization in making decisions on specific positions.

## Data availability statement

The raw data supporting the conclusions of this article will be made available by the authors, without undue reservation.

## Ethics statement

The studies involving humans were approved by the Ethics Committee of Riga Stradins University. The studies were conducted in accordance with the local legislation and institutional requirements. Written informed consent for participation in this study was provided by the participants' legal guardians/next of kin.

## Author contributions

IJ: Data curation, Formal analysis, Methodology, Resources, Writing – original draft, Writing – review & editing. IT-K: Data curation, Formal analysis, Methodology, Writing – review & editing. JL: Conceptualization, Formal analysis, Investigation, Supervision, Writing – review & editing. IM: Supervision, Writing – review & editing. JK: Conceptualization, Data curation, Investigation, Methodology, Project administration, Supervision, Writing – review & editing.

## References

[ref9001] Amthauer R. (1973). Intelligenz-Struktur-Test LS. T. 70. Handan-weisung fair die durchfiihrung und auswertung. Gottingen: Verlag fur Psychologie. Eds. Amthauer, R., Brocke, B., Liepmann, D., and Beauducel, A. Intelligenz-Struktur-Test 2000 (I-S-T 2000). Göttingen: Hogrefe.

[ref1] Annan-Brew R. K. (2021). Sensitivity of DIFAS, jMetrik and STATA software in determining gender DIF using MH procedure. Int. J. Res. Eng. Technol. 6, 1–24. doi: 10.29126/24570060

[ref2] Albano A. (2018). Introduction to educational and psychological measurement using R. Available at: http://www.thetaminusb.com/intro-measurement-r/index.html#license

[ref3] Beauducel A. Liepmann D. Brocke B. Amthauer R. (2010). Intelligence structure test: English version of the Intelligenz-Struktur-test 2000 R I-S-T 2000 R. Göttingen: Hogrefe.

[ref4] Bejar I. I. (1983). Achievement testing: Recent advances. Beverly Hills, CA: Sage.

[ref5] Bichi A. A. (2015). Item analyses using derived science achievement test data. Int. J. Sci. Res. 4, 1655–1662.

[ref6] Bühner M. Ziegler M. Krumm S. Schmidt-Atzert L. (2006). Ist der I-S-T 2000 R Rasch-skalierbar? [is the I-S-T 2000 R Rasch scaleable?]. Diagnostica 52, 119–130. doi: 10.1026/0012-1924.52.3.119

[ref8] Carroll J. B. (1993). Human cognitive abilities: A survey of factor analytic studies. Cambridge: Cambridge University Press.

[ref9] Cattell R. B. (1940). Culture fair intelligence test (CFIT) [database record]. APA PsycTests. doi: 10.1037/t14354-000

[ref10] Cattell R.B. (1987). Intelligence: Its structure, growth, and action. Amsterdam: Elsevier Science Publishers B. V.

[ref9003] Chen F. F. (2007). Sensitivity of goodness of fit indexes to lack of measurement invariance. Structural Equation Modeling, 14, 464–504. doi: 10.1080/10705510701301834

[ref11] Dragūnevičius K. Gintilienė G. (1998). Lithuanian I-S-T version: reliability and validity. Psichologija 18, 47–64. doi: 10.15388/Psichol.1998.4450

[ref12] Embretson S. E. Reise S. P. (2000). Item response theory for psychologists. Mahwah, NJ: Lawrence Erlbaum.

[ref13] Field A. (2017). Discovering statistics using SPSS (5th ed.). London, UK: Sage Publications Ltd.

[ref14] Guilford J. R. (1967). The nature of human intelligence. New York: McGraw-Hill.

[ref15] Guttman L. (1965). “A faceted definition of intelligence” in Studies in psychology. Scripta Hierosolymitana. ed. Eiferman R., vol. 14 (Jerusalem: The Hebrew University), 166–181.

[ref19] Horn J. L. Cattell R. B. (1966). Refinement and test of the theory of fluid and crystallized general intelligences. J. Educ. Psychol. 57, 253–270. doi: 10.1037/h0023816, PMID: 5918295

[ref20] Kaplan R. M. Saccuzzo D. P. (2009) Psychological testing principles, applications, and issues (7th Edn.). Belmont, CA: Wadsworth, Cengage Learning.

[ref21] Kline P. (1999). The handbook of psychological testing (2nd ed.). London: Routledge.

[ref22] Lange P. C. (1967). Future Developments. Teach. Coll. Rec. 68, 284–325. doi: 10.1177/016146816706801010

[ref23] van Leeuwen M. van den Berg S. M. Boomsma D. I. (2008). A twin-family study of general IQ. Learn. Individ. Differ. 18, 76–88. doi: 10.1016/j.lindif.2007.04.006

[ref24] Liepmann D. Beauducel A. Brocke B. Amthauer R. (2007). Intelligenz-Struktur-Test 2000 R (I-S-T 2000 R). Göttingen: Hogrefe Verlag GmbH & Co.

[ref25] Ļubenko J. Jokste I. Trups-Kalne I., un Koļesņikova, J. (2022). Intelekta struktūras tests: (I-S-T 2000). R latviešu valodas versija: Rokasgrāmata. Rīga: Rīgas Stradiņa universitāte.

[ref27] Marcoulides K. M. Yuan K.-H. (2017). New ways to evaluate goodness of fit: a note on using equivalence testing to assess structural equation models. Struct. Equ. Model. 24, 148–153. doi: 10.1080/10705511.2016.1225260

[ref28] McDonald R. P. (1999). Test theory: a unified treatment. New York: Lawrence Erlbaum Associates Publishers.

[ref29] Medvedev O. N. Krägeloh C. U. (2022). “Rasch Measurement Model” in Handbook of assessment in mindfulness research. eds. Medvedev O. N. Krägeloh C. U. Siegert R. J. Singh N. N. (Cham: Springer) doi: 10.1007/978-3-030-77644-2_4-1

[ref30] Meyer J. P. (2014). Applied measurement with jMetrik. London: Routledge.

[ref31] O'Connor T. R. Eskey M. T. (2005). “Types of scales and indexes” in Encyclopedia of social measurement. ed. Kempf-Leonard I. K. (Elsevier), 443–453.

[ref32] Olkin I. Sampson A. R. (2001). “Multivariate analysis: overview. In Neil J. Smelser, N.J.” in Paul B. Baltes, P.B. (Eds.) international encyclopedia of the social & behavioral sciences. Pergamon, 10240–10247. doi: 10.1016/B0-08-043076-7/00472-1

[ref33] Pellerone M. Passanisi A. Bellomo M. F. (2015). Identity development, intelligence structure, and interests: a cross-sectional study in a group of Italian adolescents during the decision-making process. Psychol. Res. Behav. Manag. 8, 239–249. doi: 10.2147/PRBM.S88631, PMID: 26316831 PMC4548755

[ref34] Penfield R. D. (2013). “Item analysis” in eds. Geisinger K. F. Bracken B. A. Carlson J. F. Hansen J.-I. C. Kuncel N. R. Reise S. P., APA handbook of testing and assessment in psychology, Vol. 1. Test theory and testing and assessment in industrial and organizational psychology, American Psychological Association. 121–138. doi: 10.1037/14047-007

[ref35] Putnick D. L. Bornstein M. H. (2016). Measurement invariance conventions and reporting: the state of the art and future directions for psychological research. Dev. Rev. 41, 71–90. doi: 10.1016/j.dr.2016.06.004, PMID: 27942093 PMC5145197

[ref36] Roid G. H. (2003). Standford-Binet intelligence scales. 5th Edn (SB:V). Itasca, IL: Riverside Publishing. Canadian Journal of School Psychology, 19, 235–244. doi: 10.1177/082957350401900113

[ref37] Sass D. A. Schmitt T. A. (2013). “Testing measurement and structural invariance” in Handbook of quantitative methods for educational research. ed. Teo T. (Rotterdam: Sense Publishers)

[ref38] Shou Y. Sellbom M. Chen H.-F. (2022). “Fundamentals of measurement in clinical psychology” in Comprehensive clinical psychology. eds. Gordon J. G. Asmundson G. J. G.. 2nd ed (Elsevier), 13–35. doi: 10.1016/B978-0-12-818697-8.00110-2

[ref39] Sternberg R. (2020). “A history of research on intelligence: part 2: psychological theory, research, and practice in the nineteenth and twentieth centuries” in The Cambridge handbook of intelligence. ed. Sternberg R. (Cambridge: Cambridge University Press), 31–46.

[ref40] Strauss E. Sherman E. M. S. Spreen O. (2006). A compendium of neuropsychological tests: Administration, norms, and commentary. 3rd Edn. Oxford: Oxford University Press.

[ref42] Šimelionienė A. Gintilienė G. (2011). Intelligence structure of 16–18 years old intellectually gifted students. Psichologija 44, 42–56. doi: 10.15388/Psichol.2011.44.2549

[ref43] Tavakol M. Wetzel A. (2020). Factor analysis: a means for theory and instrument development in support of construct validity. Int. J. Med. Educ. 11, 245–247. doi: 10.5116/ijme.5f96.0f4a, PMID: 33170146 PMC7883798

[ref44] Thurstone L. L. (1947). Multiple factor analysis development and expansion of the vectors of mind. Chicago: University of Chicago Press

[ref45] Van der Elst W. Ouwehand C. van Rijn P. Lee N. Van Boxtel M. Jolles J. (2013). The shortened raven standard progressive matrices: item response theory-based psychometric analyses and normative data. Assessment 20, 48–59. doi: 10.1177/1073191111415999, PMID: 21807748

[ref46] Van Tassel-Baska J. (2005). Gifted programs and services: what are the nonnegotiables? Theory Pract. 44, 90–97. doi: 10.1207/s15430421tip4402_3

[ref48] Verguts T. De Boeck P. (2000). A Rasch model for detecting learning while solving an intelligence test. Appl. Psychol. Meas. 24, 151–162. doi: 10.1177/01466210022031589

[ref49] Vernon P. E. (1961). The structure of human abilities. London: Methuen

[ref50] Wechsler D. (1997). Wechsler adult intelligence scale–third edition (WAIS-III) [database record]. APA PsycTests. doi: 10.1037/t49755-000

[ref51] Woodcock R. W. McGrew K. S. Mather N. (2001). Woodcock–Johnson III tests of cognitive ability. Itasca, IL: Riverside Publishing.

[ref52] Ziegler M. Hagemann D. (2015). Testing the Unidimensionalityof items: pitfalls and loopholes. Eur. J. Psychol. Assess. 31, 231–237. doi: 10.1027/1015-5759/a000309

