# Supplementary material for: Adaptation of the Intelligence Structure Test, Latvian version: psychometric properties
Source: Front Psychol. 2024 Mar 19;15:1319983. doi: 10.3389/fpsyg.2024.1319983 (PMC10985252; doi:10.3389/fpsyg.2024.1319983)
Supplement: Supplementary file 1 [file Data_Sheet_1.docx]

**Appendices**

~~
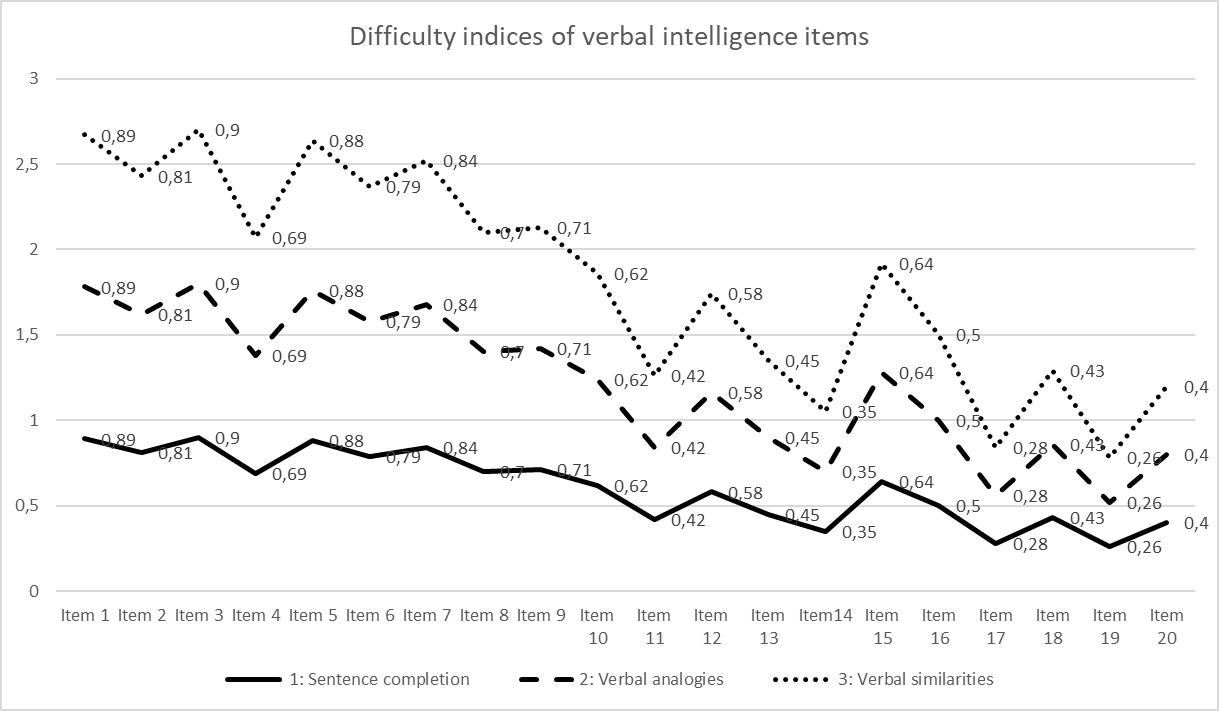
~~

*Figure 1. Difficulty indices of verbal reasoning items*

~~
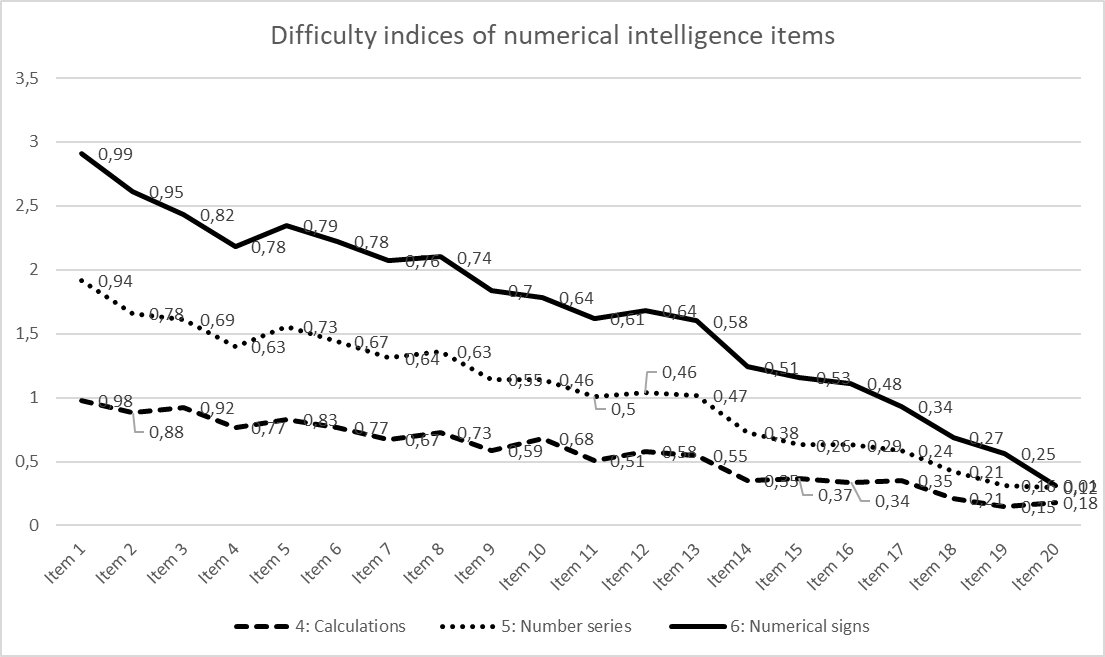
~~

*Figure 2. Difficulty indices of numerical reasoning items.*

~~
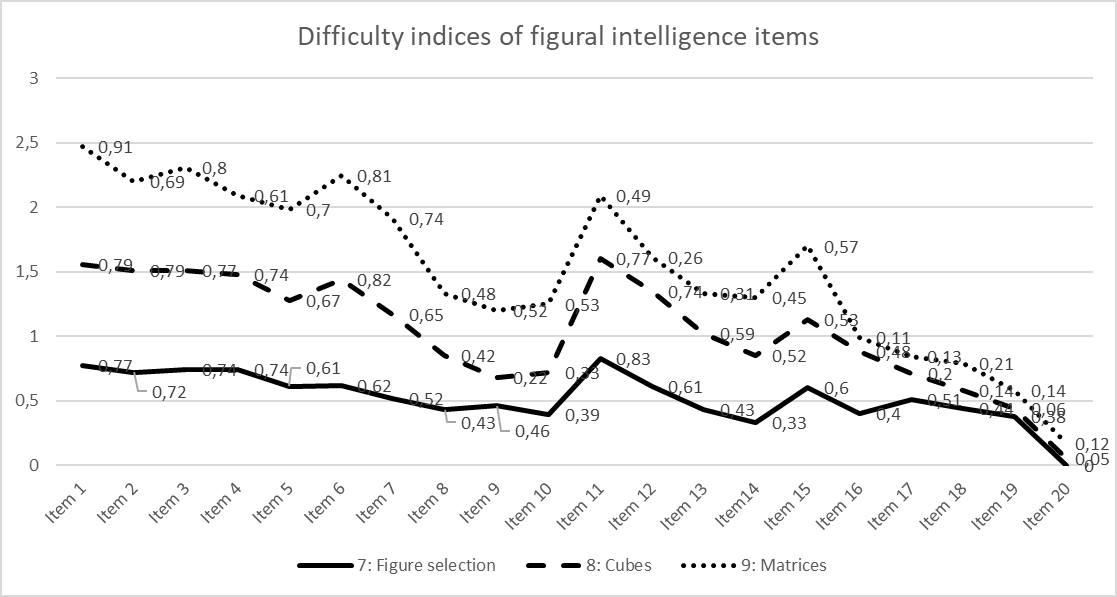
~~

*Figure 3. Difficulty indices of the figural reasoning items*
